# Data on the observation of the interface between lithium phosphorus oxynitride film and lithium layer

**DOI:** 10.1016/j.dib.2020.106612

**Published:** 2020-12-01

**Authors:** Jaehwan Ko, Young Soo Yoon

**Affiliations:** Department of Materials Science and Engineering, Gachon University, Seongnam, Republic of Korea

**Keywords:** Lithium phosphorus oxynitride, Lithium protective layer, Lithium metal batteries, Lithium dendrite, Cross-sectional images

## Abstract

This data provides the observation of the interface between lithium phosphorus oxynitride(LiPON) film and lithium layer. In other words, the shape of the lithium electrode protected by the LiPON film after the dissolution/precipitation cycle is provided as images. Also, for clear interpretation of these images, the shape of lithium formed between LiPON and copper current collector is provided as images. Readers are requested to go through the article entitled “Suppression of formation of lithium dendrite via surface modification by 2-D lithium phosphorus oxynitride as a highly stable anode for metal lithium batteries” (Ko et al., 2020) for further interpretation and discussion. Since the article provided only surface images for the above-mentioned items, it is expected that the cross-sectional images provided in this data will help readers to understand the overall contents in depth.

**Specifications Table**

**Table:** 

Subject	Materials Science
Specific subject area	Energy material, thin film process and electrochemistry
Type of data	Image
How data were acquired	FE-SEM (S-4200 system, Hitachi), Battery cycler (WBCS 3000, Wonatech), CR2032 coin-type cell, RF-sputtering system
Data format	Raw, Analyzed
Parameters for data collection	Internal structure of coin-type cell, LiPON film formation conditions, Cycle condition of Li symmetric cell: 1 mA/cm^2^ and 1 mAh/cm^2^ (50 cycles), Electrodeposition condition of Li│Cu cell: (1 mA/cm^2^ or 2 mA/cm^2^)and 1 mAh/cm^2^
Description of data collection	LiPON deposition on Li foil or Cu foil using RF-sputtering, Sample preparation using CR2032 coin-type cell, Sectional sample preparation using FIB, Observation of sample cross section using FE-SEM
Data source location	Gachon University, Seongnam, Republic of Korea
Data accessibility	With the article
Related research article	J. Ko, D. H. Cho, D. J. Kim, Y. S. Yoon, Suppression of formation of lithium dendrite via surface modification by 2-D lithium phosphorus oxynitride as a highly stable anode for metal lithium batteries, J. Alloy. Compd. 845 (2020) 156280. https://doi.org/10.1016/j.jallcom.2020.156280

## Value of the Data

• These data provide useful information on the Li dendrite inhibitory effect of LiPON film and Li formation at the interface.
• These data provides useful information for researchers interested in the practical use of metal lithium batteries, especially lithium protective layers.
• Based on these data, it is possible to further study the uniform and defect-free LiPON protective film and the behavior of the Li layer according to the cycle.

## 1 Data Description

**Fig. 1:**
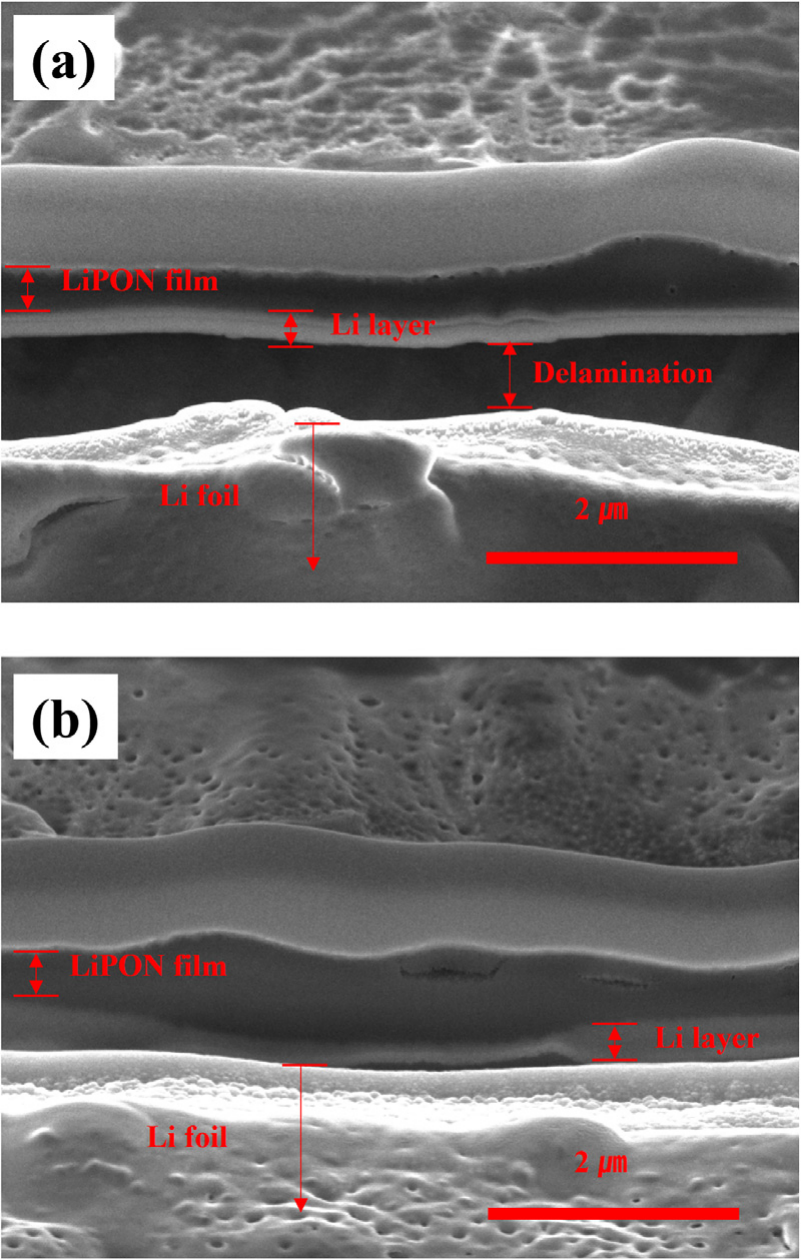
Cross-sectional SEM images of the electrode after 50 cycles (1 mA/cm^2^, 1 mAh/cm^2^) of Li symmetric cell with LiPON protective layer; (a) severe delamination, (b) slight delamination.

Fig. 1 shows the SEM images of the cross-sectional state of LiPON-coated Li electrode after 50 cycles (1 mA/cm^2^ and 1 mAh/cm^2^) of symmetric cells. After cycling, the boundary between the LiPON film and Li layer is more clearly observed. This is because the Li layer formed due to the repeated dissolution/precipitation reaction of Li during cycling shows a heterogeneous shape different from the other layers (LiPON film, Li foil.) The LiPON film of about 200 to 300 nm thick was maintained after 50 cycles, and a Li layer, with a thickness of approximately 200 to 300 nm that participated in the dissolution/precipitation reaction, was formed under the LiPON film.

**Fig. 2:**
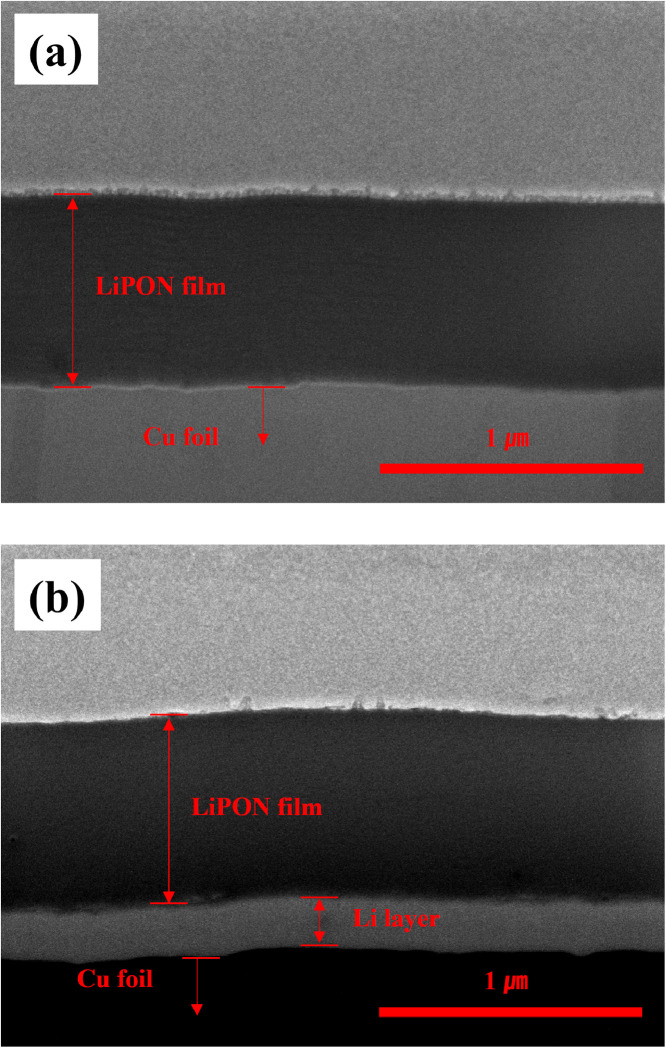
Cross-sectional SEM images of the electrode of Li│Cu cell with LiPON protective layer; (a) before Li electrodeposition, (b) after Li electrodeposition (1 mA/cm^2^, 1 mAh/cm^2^).

**Fig. 3:**
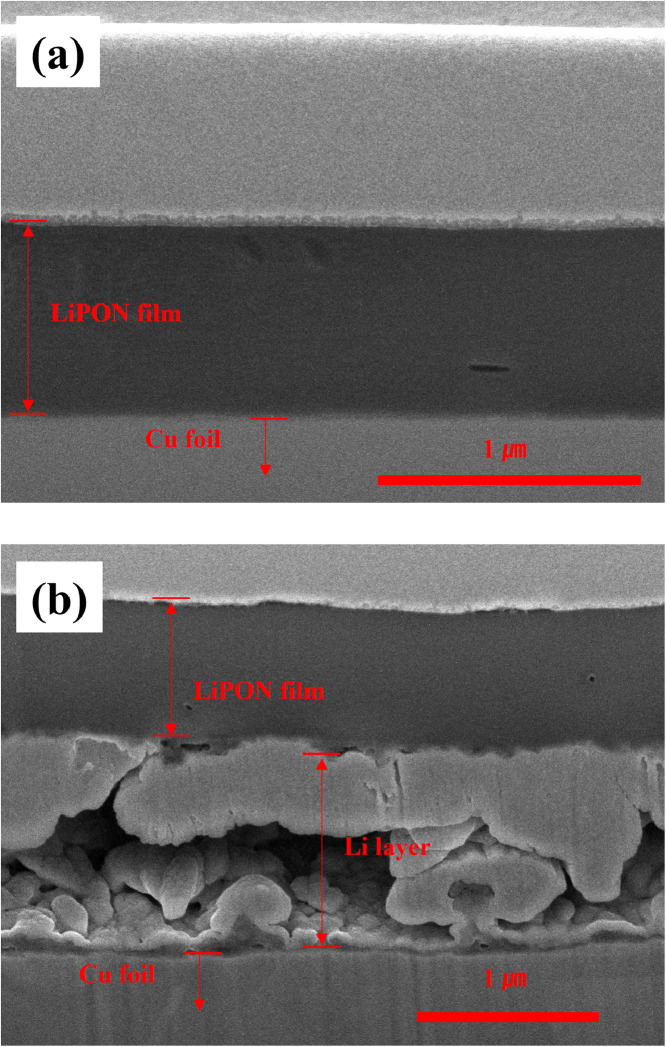
Cross-sectional SEM images of the electrode of Li│Cu cell with LiPON protective layer; (a) before Li electrodeposition, (b) after Li electrodeposition (2 mA/cm^2^, 1 mAh/cm^2^).

However, both LiPON and Li are sensitive to electron beams, making detailed observation difficult and blurring of the boundaries between the materials. The cracks and delamination observed are traces of damage to the electron beam. For clear interpretation of these images, Fig. 2, Fig. 3 show the shape of Li formed between LiPON and Cu current collector. In Fig. 2, Li layer was electrodeposited on the interface between LiPON and copper current collector under the condition of 1 mA/cm^2^ and 1 mAh/cm^2^. The thickness of the layer is 240 nm, which is similar to the Li layer in Fig. 1. In Fig. 3, Li layer was electrodeposited on the interface between LiPON and copper current collector under the condition of 2 mA/cm^2^ and 1 mAh/cm^2^. A Li layer having a thickness of about 1.5 *μ*m was formed due to the effect of the doubled current density, but the state of the layer was very non-uniform and a low-density layer having many pores was shown.

## 2 Experimental Design, Materials and Methods

All reagents purchased were of analytical grade and were used as received. LiPON films were deposited by RF sputtering from a Li_3_PO_4_ target (purity: 99.99%, Umicore, Belgium Brussels.) A lithium foil with 200 *μ*m thickness and a copper foil with 50 *μ*m thickness were used as substrates under argon atmosphere, inside a glove box. Because the glove box is connected with a sample-loading chamber of the sputtering system, there was no side reaction when the Li foil was loaded in the sputtering chamber. Before each deposition, 30 min pre-sputtering was performed under argon atmosphere. LiPON layers were deposited at a nitrogen pressure of 0.66 Pa and a RF power density of 2.4 W/cm^2^.

The Li│Li symmetric cell was fabricated to evaluate the LiPON-coated Li anode using CR2032 coin cell in an argon atmosphere (Purity: 99.999%) inside a glove box. The coin cells were assembled using LiPF_6_ in EC: DEC (1:1) as the electrolyte and a polypropylene sheet as a separator. The amount of electrolyte was 20 μl in a coin cell. Li electrodes were prepared with an electrode area of 0.5 cm^2^. The Li│Li symmetric cell was cycled at 1 mA/cm^2^ and 1 mAh/cm^2^ during 50 cycles, and the cross-sectional images of the LiPON-coated Li electrode were obtained from the cycled cell. The cycled cell was decomposed in an argon atmosphere inside a glove box, and the collected LiPON-coated Li electrode was rinsed with DEC and then analysed. The Li│Cu cells were fabricated to confirm the shape of Li layer participating in dissolution/precipitation reaction using CR2032 coin cell in an argon atmosphere (Purity: 99.999%) inside a glove box. The coin cells were assembled using LiPF_6_ in EC: DEC (1:1) as the electrolyte and a polypropylene sheet as a separator. The amount of electrolyte was 20 μl in a coin cell. Li and Cu electrodes were prepared with an electrode area of 0.5 cm^2^. Li layers were electrodeposited on the interface between LiPON and copper current collector under the condition of (1 mA/cm^2^ or 2 mA/cm^2^) and 1 mAh/cm^2^. The cross-sectional images of the LiPON/electrodeposited Li/Cu electrode were obtained from the Li│Cu cells. The cells were decomposed in an argon atmosphere inside a glove box, and the collected electrodes were rinsed with DEC and then analysed.

The cross sectional morphology of the electrode was characterized using field emission scanning electron microscopy (FE-SEM, Hitachi S-4200 system, Japan). The dissolution/precipitation cycle and electrodeposition were performed using a battery cycler at 25 °C (WBCS 3000, Wonatech Co. Ltd., Korea) [1].

## CRediT Author Statement

**Jaehwan Ko:** Conceptualization, Methodology, Writing- Original draft preparation. **Young Soo Yoon:** Writing- Reviewing and Editing, Supervision.

## Declaration of Competing Interest

The authors declare that they have no known competing financial interests or personal relationships which have or could be perceived to have influenced the work reported in this article.
